# Registry on extracorporeal multiple organ support with the advanced organ support (ADVOS) system: 2-year interim analysis

**DOI:** 10.1097/MD.0000000000024653

**Published:** 2021-02-19

**Authors:** Valentin Fuhrmann, Aritz Perez Ruiz de Garibay, Andreas Faltlhauser, Bartosz Tyczynski, Dominik Jarczak, Jens Lutz, Julia Weinmann-Menke, Andreas Kribben, Stefan Kluge

**Affiliations:** a Universitätsklinikum Hamburg-Eppendorf, Klinik für Intensivmedizin, Hamburg, Deutschland; b Universitätsklinikum Münster, Medizinische Klinik B für Gastroenterologie and Hepatologie, Münster; c Evangelisches Krankenhaus Duisburg-Nord, Klinik für Innere Medizin, Duisburg; d ADVITOS GmbH, Munich; e Klinikum Weiden, Medizinische Klinik 1, Weiden; f Universitätsklinikum Essen, Klinik für Nephrologie, Essen; g Gemeinschaftsklinikum Mittelrhein, Innere Medizin Nephrologie-Infektiologie, Koblenz; h Universitätsmedizin Mainz, I. Medizinische Klinik and Poliklinik, Mainz, Germany.

**Keywords:** advanced organ support, albumin dialysis, extracorporeal organ support, multiple organ failure, multiple organ support therapy, real world evidence

## Abstract

The objective of this registry is to collect data on real-life treatment conditions for patients for whom multiple organ dialysis with Advanced Organ Support (ADVOS) albumin hemodialysis is indicated.

This registry was performed under routine conditions and without any study-specific intervention, diagnostic procedures, or assessments. Data on clinical laboratory tests, health status, liver function, vital signs, and examinations were collected (DRKS-ID: DRKS00017068). Mortality rates 28 and 90 days after the first ADVOS treatment, adverse events and ADVOS treatment parameters, including treatment abortions, were documented.

This analysis was performed 2 years after the first patient was included on January 18, 2017. As of February 20, 2019, 4 clinical sites in Germany participated and enrolled 118 patients with a median age of 60 (IQR: 45, 69) of whom 70 were male (59.3%). Patients had a median SOFA Score of 14 (IQR: 11, 16) and a predicted mortality of 80%. The median number of failing organs was 3 (IQR: 2, 4).

Four hundred twenty nine ADVOS treatments sessions were performed with a median duration of 17 hours (IQR: 6, 23). A 5.8% of the ADVOS sessions (25 of 429) were aborted due to device related errors, while 14.5% (62 of 429) were stopped for other reasons. Seventy nine adverse events were documented, 13 of them device related (all clotting, and all recovered without sequels).

A significant reduction in serum creatinine (1.5 vs 1.2 mg/dl), blood urea nitrogen (24 vs 17 mg/dl) and bilirubin (6.9 vs 6.5 mg/dl) was observed following the first ADVOS treatment session. Blood pH, bicarbonate (HCO_3_^-)^ and base excess returned to the physiological range, while partial pressure of carbon dioxide (pCO_2)_ remained unchanged. At the time of the analysis, 28- and 90-day mortality were 60% and 65%, respectively, compared to an expected ICU-mortality rate of 80%. SOFA score was an independent predictor for outcome in a multivariable logistic regression analysis.

The reported data show a high quality and completion of all participating centers. Data interpretation must be cautious due to the small number of patients, and the nature of the registry, without a control group. However, the data presented here show an improvement of expected mortality rates. Minor clotting events similar to other dialysis therapies occurred during the treatments.

## 1 Introduction

The mortality rate of patients with multiple organ failure is high, despite the improvement in the management of critically ill patients.^[1,2]^ Acute kidney injury, liver injury and respiratory failure might be present in more than 50% of the patients in the intensive care unit (ICU), either alone or in various combinations.^[3–6]^ In fact, a simultaneous multiple organ failure (MOF) can lead to mortality rates over 80% in patients with Sequential Organ Failure Assessment (SOFA) score of 14 (i.e., ≥4 organ failure) or higher.^[7–10]^

Currently, the term extracorporeal organ support (ECOS) is being employed to describe all forms of therapies entailing blood extraction and further processing it in specifically designed circuits and devices.^[11]^ Tissues from affected organs are all perfused with blood, which results in the main target for these treatments.^[12]^ As such, therapies including toxin removal, correction of acid-base disturbances, balance of electrolyte and fluid, carbon dioxide (CO_2_) removal and, even oxygenation should be part of a unified system. Instead, modular hardware as add-on devices to a continuous renal replacement therapy (CRRT) circuit is currently common.^[13,14]^ Since there is often a relation between an organ dysfunction and the impairment of other organs, a multidisciplinary management with a focus on MOF is needed.^[15]^

The Advanced Organ Support (ADVOS) therapy integrates kidney, liver, and lung support in 1 single device. In this ECOS procedure, a recirculating and recyclable albumin enriched solution serves as the primary dialysate fluid and is intended to remove protein-bound toxins from the blood. Thereby, in contrast to conventional dialysis procedures, not only water-soluble substances (e.g., creatinine, urea, and ammonia) are eliminated, but also albumin-bound substances (e.g., bilirubin, bile acids, aromatic amino acids, copper) can be removed, as shown in preclinical and clinical studies.^[16–19]^ Additionally, thanks to a revolutionary dialysate recirculating and recycling circuit, the pH value and composition of the dialysate can be individualized for each patient, which allows acid-base balance control, including metabolic acidosis correction and CO_2_ removal.^[20–22]^

We present here the objectives and design of a noninterventional, multi-center, and nonrandomized patient registry. The objective of the study was to collect data on real-life treatment conditions for patients for whom multiple organ dialysis with the ADVOS device was indicated without any study-specific intervention, diagnostic procedures, or assessments. Collected data comprised safety and performance parameters, including outcome indicators (i.e., 28- and 90-day mortality). The criteria used to analyze safety and performance is depicted in Table 1 and described in detail in the following section. This report summarizes and discusses the data obtained during the 2 years after the inclusion of the first patient.

**Table 1:** Performance and safety criteria.

Performance	Safety
Mortality rates 28 and 90 days after the first treatment session.	Vital signs;
Chronic liver failure-sequential organ failure assessment (CLIF-SOFA), sequential organ failure assessment (SOFA), and quick SOFA (qSOFA) scores;	Laboratory (including blood chemistry, hematology, coagulation, and urine);
Severity of liver disease assessed by the Model for End-Stage Liver Disease (MELD) score;	Adverse events;
Child-Pugh Score;	Assessment of breathing;
Simplified Acute Physiology (SAPS) Score II;	Blood gas analysis, including base excess
Cardiac output and other hemodynamic parameters;	
Indocyanine green dye (ICG) clearance;	
Occurrence of ascites and hepatic encephalopathy;	
Biochemical tests reflecting organ damage, such as total bilirubin, albumin, and creatinine;	

## 2 Objectives and design

This was a non-interventional, multi-center, non-randomized registry in post marketing surveillance. The objective was to collect data on real-life treatment conditions for patients for whom multiple organ dialysis is indicated without any study-specific intervention, diagnostic procedures, or assessments. This information should help to improve the treatment of these seriously ill patients in the future.

In this registry, the hemodialysis device ADVOS multi, including applicable accessories (i.e., fluid processing units), and fluids (i.e., alkaline concentrate, acidic concentrate, Dia Protect, 20% sodium chloride concentrate) was used for extracorporeal multiple organ dialysis to support liver and kidney function which is in line with the intended use being defined for this medical device in accordance with its CE mark.

The registry evaluated the performance and safety of the procedure and recorded mortality rates at 28 and 90 days. The registry was intended to help to create recommendations for ADVOS treatments as well as to identify appropriate supportive and diagnostic measures. Finally, it was evaluated if registry data on adverse events, mortality rates and treatment under real-life practice conditions were comparable with published data from other trials.

### 2.1 The ADVOS system

The ADVOS multi (ADVITOS GmbH, Munich, Germany) is a hemodialysis device indicated for patients with acute, chronic, and acute-on-chronic liver failure or renal failure. It is especially intended to remove water-soluble and protein-bound toxic substances, to normalize or improve composition of blood in case of electrolyte or acid-base disturbances, and to remove fluids in case of fluid overload.

**Figure 1:**
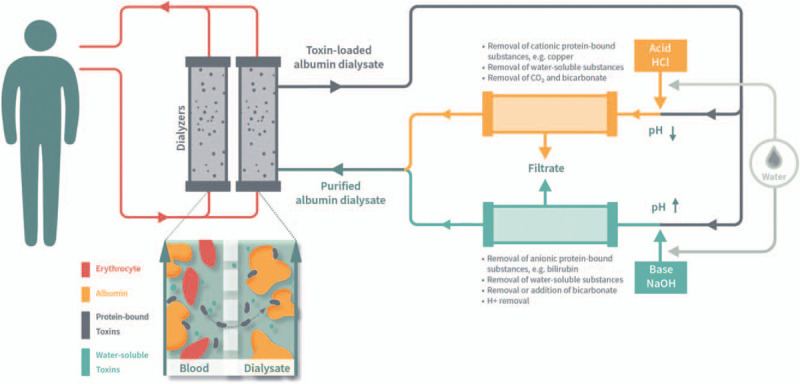
Schematic representation of the ADVOS system.

Briefly, patients are connected to the blood tubing set of the ADVOS multi through a conventional double lumen dialysis catheter (e.g., 13 F diameter). The patient blood is then cleaned by passing it through two parallel high-flux dialyzers with a 1.9 m^2^ effective surface (Fig. 1). ADVOS multi runs at low blood flows between 100 and 400 ml/minutes. ADVOS sessions might be intermittent or continuous up to 24 hours. Anticoagulation is strongly recommended during treatments and was employed on clinical judgement. Data on the type of anticoagulation was not recorded for this registry. However, common modalities normally include unfractionated heparin, regional citrate anticoagulation or thrombin inhibitors, as described elsewhere.^[19,20]^

The dialysate results from mixing an alkaline concentrate (i.e., mainly NaOH), an acidic concentrate (i.e., mainly HCl) and osmosis water. It consists of sodium, chloride, potassium, calcium, magnesium, phosphate, and bicarbonate. It contains 200 ml of 20% pharmaceutical grade albumin, which is sourced from the hospital's pharmacy. The ADVOS multi employs a recirculating procedure for the dialysate. For this purpose, the dialysate is divided into 2 paths, where the pH level is either increased (i.e., addition of alkaline concentrate) or decreased (i.e., addition of acidic concentrate) to release anionic (e.g., bilirubin) or cationic (e.g., copper) substances from albumin, respectively. This allows to restore the dialysate albumin binding capacity and to filter toxins by convection through 2 high-flux filters with an effective surface of 1.3 m^2^.^[20,21]^ The removed filtrate is continuously replaced by permeate (i.e., osmosis water) and alkaline and acidic concentrates.

By adjusting the ratio of the alkaline and acidic concentrates, the pH of the dialysate can be adjusted within its ranges (i.e., 7.2–9.0). Concentrate flows can be adjusted between 160 and 320 ml/minutes. All added and replaced fluids are continuously balanced, including the user-defined ultrafiltration (UF) rate. The permeate is provided in the movable container under the machine. In this container, the used filtrate (waste liquids) is accumulated in a separate bag.

The ADVOS multi and its fluids are not intended for children, pregnant or nursing women, or persons with Creutzfeldt-Jakob disease.

### 2.2 Study population

This interim analysis was performed 2 years after the first patient was included on January 18, 2017. Adult patients (i.e., ≥18 years) requiring Advanced Organ Support according to the indications for use (see above) and at discretion of the treating physician, were enrolled in the registry. No exclusion criteria was defined.

As of February 20, 2019, 4 clinical sites in Germany participated and enrolled 118 patients. In detail, the University Hospital Hamburg-Eppendorf (UKE) enrolled 79 patients, the Mainz University Medical Center from the Johannes Gutenberg University, included 7 patients, the University Hospital in Essen registered 13 patients and the Weiden Clinic (Kliniken Nordoberpfalz AG) contributed with 19 patients.

### 2.3 Data documentation

Data on clinical laboratory tests, vital signs, health status, liver function, and ADVOS multi treatment parameters were collected at different time points:

- At hospital admission
- At baseline (i.e. immediately before the first ADVOS treatment session)
- After the first ADVOS treatment session
- On Days 1, 3, and 7 after the first ADVOS treatment session, and once immediately after the last session of the same treatment cycle (i.e., with ≤1 week of treatment interruption).
- On days 28 and 90 after the first treatment session.

If due to a worsening of the health condition during the observation period, a new ADVOS session was deemed appropriate on an enrolled patient who was not treated with ADVOS for >1 week, the patient was considered a new subject. This strategy pretended to homogenize the baseline characteristics of the patients. However, the patient was considered as a single subject for mortality rate analysis. Only 2 patients were on this situation.

All patients who were exposed to the registry medical device were evaluated for Adverse Events (AEs) like catheter problems, bleeding, allergic reactions, clotting, electrolyte imbalances, and infections.

### 2.4 Data management and record keeping

A fully web-based Trial software consisting of an Electronic Data Capturer (EDC), a Clinical Trial Management System (CMTS) and a Safety Control Center (SCC) was employed for data handling (CT-Engine. Trium Analysis Online GmbH, Munich, Germany).

Monitoring, project management, and data management was done by FGK Clinical Research (Munich, Germany) following standard operating procedures. FGK Clinical Research handled the data cleaning process, including logical check, and query processes.

All required clinical data for this registry study were collected with an electronic Case Report Form (eCRF). Consistency checks were applied to verify correctness of data. An audit trail recorded all entries and corresponding changes.

For patients who were unable or unwilling to provide informed consent and for patients whose data were collected retrospectively, data was anonymous (i.e., untraceable) and could not be associated to a patient's name.

### 2.5 Criteria for evaluation

No specific primary or secondary endpoint was defined, as the objective was simply to collect data on real-life conditions during multiple organ dialysis. To evaluate the performance and safety, the assessments depicted in Table 1 were performed.

The settings (i.e., blood flow, dialysate pH, treatment duration…) employed during ADVOS treatments were summarized for each patient after the last treatment session was conducted.

### 2.6 Statistics and sample size

Continuous variables are reported as mean and standard error. Shapiro–Wilk and Levene tests were performed to assess the normal distribution of samples and the homogeneity of variance, respectively. The Student *t* test for paired samples was used to compare values before and after ADVOS sessions and treatments. Variables without homogeneous distribution were compared via Mann–Whitney *U* Test. A two-tailed *P* value lower than .05 was considered to indicate statistical significance. Mortality was assessed using Kaplan–Meier curves. Data were analyzed with IBM SPSS 24.0 for Windows. No statistical comparison was performed for binary variables.

A multivariable logistic regression model was developed to evaluate patient demographic and baseline clinical parameters (immediately before the first ADVOS session) associated with death, as previously done in other patient registries.^[23]^ Briefly, variables that were statistically associated with mortality in a bivariate analysis were considered as candidate variables in the multivariable model. A forward step-wise selection procedure with inclusion and exclusion cut-offs of 0.05 and 0.10, respectively, was followed. Adjusted odds ratios with 95% confidence intervals were reported. The cases with missing data were excluded. The Cox and Snell, and the Nagelkerke statistics were reported as a measure of variability explained by the model.

### 2.7 Ethical principles, patient safety, data protection and funding

This study was approved by the Bavarian State Medical Association (Bayerische Landesärztekammer) on the 2nd of November 2016 and is registered in the German Registry for Clinical Studies (DRKS) and the International Clinical Trials Registry Platform from the World Health Organization (DRKS00017068). The registry poses no additional risks to the patient beyond those related to the collection and storage of the data. The patients were treated on-label, and their treating physician had already made the decision that treatment with a multiple organ dialysis was indicated.

All patients who participated in the registry and for whom pseudonymous data collection was scheduled had to sign an informed consent form. For patients who did not consent, no personal registry-related data can be accessed due to the untraceable nature of the anonymous data.

Personal patient data is kept confidential in compliance with the European Data Protection Directive and other applicable international and national requirements.^[24]^ The necessary amendments were performed during the conduction of this registry in order to adapt to the new EU General Data Protection Regulation.^[25]^

The costs for the registry study management and ethics committee fees were borne by ADVITOS GmbH.

## 3 Results

### 3.1 Baseline characteristics

**Figure 2:**
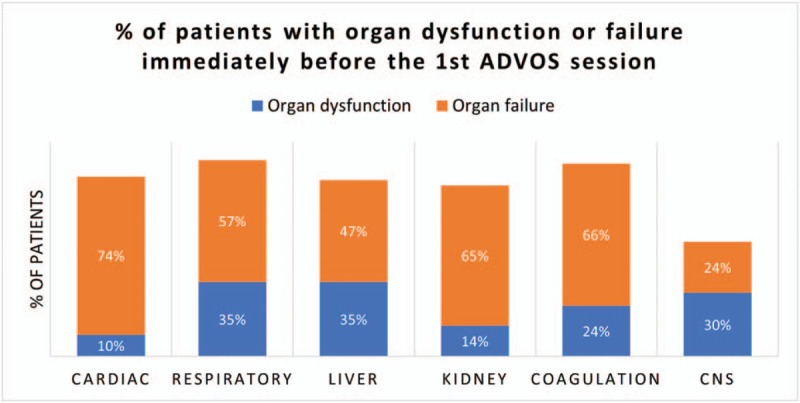
Percentage of patients with organ dysfunction or failure immediately before the 1st ADVOS session.

Median age of registry participants was 60 years (IQR: 45, 69), and 70 of them were male (59.3%). Patients were critically ill with a median SOFA Score immediately before the first ADVOS session of 14 (IQR: 11, 16) and a predicted mortality of 80%. The median number of failing organs was 3 (IQR: 2, 4) (Fig. 2). Table 2 summarizes baseline characteristics. Data on medical history at hospital admission is provided in Supplementary Table 1.

**Table 2:** Baseline characteristics of the 118 patients immediately before the 1st ADVOS treatment session. Median (IQR) or percentage.

Parameter	Before 1st ADVOS
Age [years]	60 (45, 69)
Gender [Male/Female]	59.3% / 40.7%
Vasopressors [%]	78.8%
Mechanical ventilation [%]	65.3%
Ascites [%]	30.5%
Acidemia (pH <7.35) [%]	52.5%
Hypercapnia (pCO_2_ > 45 mm Hg) [%]	25.4%
Metabolic acidosis (HCO_3_^-^ <22 mmol/l) [%]	55.1%
Glasgow Coma Score	14 (13, 15)
SOFA Score	14 (10,16)
Number of Organ dysfunction / failure^†^	1 (1, 2) / 3 (2, 4)
Liver dysfunction / failure [%]	35% / 47%
Kidney dysfunction / failure [%]	14% / 65%
Respiratory dysfunction / failure [%]	35% / 57%
Cardiovascular dysfunction / failure [%]	10% / 74%
Coagulation dysfunction / failure [%]	24% / 66%
CNS dysfunction / failure [%]	30% / 24%
MELD Score^∗^ (n = 67)	30 (24, 36)
Child-Pugh Score^∗^ (n = 57)	10 (9, 12)
qSOFA Score	2 (1, 2)

### 3.2 Performance of ADVOS

#### 3.2.1 Removal of water-soluble and protein-bound toxic substances

As shown in Table 3, a significant reduction in median serum creatinine (1.5 vs 1.2 mg/dl) and blood urea nitrogen (BUN) (24 vs 17 mg/dl) was observed during the first ADVOS treatment session. Similarly, median bilirubin levels were significantly reduced (6.9 vs 6.5 mg/dl). These values did not increase after the last ADVOS treatment session (Table 3).

**Table 3:** Course of treatment performance parameters. The analysis of patients with a completed data set before and after the first ADVOS treatment session, and after the last ADVOS treatment session is summarized. Median (IQR). Patients dead without data recording after the first ADVOS treatment session are excluded from the analysis since no pairing for different time points was possible.

Parameter	Before 1st ADVOS	After 1st ADVOS	*P* value (before-after 1st)	After Last ADVOS	*P* value (before-after last)
Bilirubin total [mg/dl]	6.9 (2.5, 18.3)	6.5 (2.4, 15.1)	<.001^∗^	7.0 (2.0 11.7)	.172
Creatinine [mg/dl]	1.5 (0.9, 2.2)	1.2 (0.7, 1.9)	<.001^∗^	1.1 (0.7, 1.6)	<.001^∗^
BUN [mg/dl]	24 (15, 38)	17 (11, 26)	<.001^∗^	17 (11, 26)	<.001^∗^
Na+ [mmol/l]	139 (136, 143)	138 (135, 141)	.011^∗^	137 (134, 141)	.002^∗^
Cl- [mmol/l]	108 (104, 111)	103 (99, 107)	<.001^∗^	105 (99, 107)	<.001^∗^
K+ [mmol/l]	4.1 (3.9, 4.7)	4.3 (4.0, 4.7)	.474	4.4 (4.1, 4.9)	.001^∗^
Calcium total [mmol/l]	2.08 (1.91, 2.22)	2.12 (1.90, 2.29)	.071	2.09 (1.88, 2.26)	.794
Magnesium [mmol/l]	0.89 (0.79, 1.03)	0.88 (0.84, 0.97)	.661	0.89 (0.80, 1.03)	.721
PaO2/FiO2	184 (145, 261)	182 (138, 266)	.417	190 (133, 264)	.633
pH	7.35 (7.26, 7.42)	7.42 (7.35, 7.46)	<.001^∗^	7.41 (7.32, 7.48)	.001^∗^
HCO3- [mmol/l]	22.1 (16.9, 25.8)	25.8 (21.8, 29.0)	<.001^∗^	24.3 (20.3, 27.5)	<.001^∗^
pCO2 [mm Hg]	38 (33, 46)	40 (33, 47)	.193	40 (32, 49)	.222
Base Excess [mmol/l]	−3.5 (−9.2, 1.1)	1.7 (−3.1, 4.8)	<.001^∗^	−0.4 (−5.5, 3.8)	<.001^∗^
Lactate [mmol/l]	3.3 (1.8, 9.9)	2.7 (1.5, 7.0)	.477	2.5 (1.6, 12.5)	.205
Haemoglobin [g/dl]	8.3 (7.5, 9.9)	8.0 (7.3, 8.7)	<.001^∗^	7.6 (7.2, 8.6)	<.001^∗^
Haematocrit [%]	24.9 (22.7, 29.3)	23.4 (21.7, 26.3)	<.001^∗^	23.1 (21.2, 25.5)	<.001^∗^
RBC [/pl]	2.6 (2.4, 3.1)	2.5 (2.3, 2.8)	.001^∗^	2.4 (2.3, 2.7)	<.001^∗^
WBC [/nl]	12.7 (6.7, 19.1)	11.7 (7.6, 19.1)	.320	11.6 (9.0, 19.8)	.880
Platelets [/nl]	81 (44, 169)	58 (30, 125)	<.001^∗^	51 (26, 95)	<.001^∗^

#### 3.2.2 Normalization and improvement of the blood composition in case of electrolyte disturbances or acid-base disorders

All the electrolytes remained in the physiological range during treatments (Table 3). A significant reduction of sodium (140 vs 138 mmol/l) and chloride (108 vs 103 mmol/l) occurred.

In fact, acid-base parameters significantly improved pH, bicarbonate (HCO_3_^-)^ and base excess returned to the physiological range, while pCO_2_ remained unchanged (Table 3).

### 3.3 ADVOS Treatment settings and safety

A total of 429 ADVOS treatments sessions were performed with a median of 3 (IQR: 1, 4) sessions per patient with a median duration of 17 (IQR: 6, 23) hours (Table 4). A 5.8% of the ADVOS sessions (25 of 429) were aborted due to device related errors, while 14.5% (62 of 429) were stopped for other reasons.

**Table 4:** ADVOS Treatment settings. Median (IQR).

Total Number of sessions	429
Duration 1st ADVOS Treatment [hour]	17 (6, 23)
Number of sessions/patient	3 (1, 4)
Session duration [hour]	16 (10, 20)
Blood flow [ml/minutes]	120 (100, 150)
Concentrate flow [ml/minutes]	160 (160, 288)
Dialysate pH	7.89 (7.40, 8.50)
UF rate [ml/hour]^∗^	212 (70, 266)
UF volume [ml]^†^	3140 (1035, 4950)

Seventy nine adverse events were documented. According to the treating physicians, 13 of them were related to the device. They were described as clotting problems and all the patients who suffered them recovered without sequels (Table 5).

**Table 5:** Adverse Events documented during ADVOS sessions.

	TOTAL	Device-related
Catheter problems	4	0
Bleeding	14	0
Allergic reaction	0	0
Clotting	29	13
Electrolyte imbalance	23	0
Infection	9	0

A significant reduction in platelet count, hemoglobin and hematocrit was observed (Table 3). However, this did not compromise the feasibility and safety of the therapy, as already shown by the small number of device-related adverse events, which occurred in only 3% of the sessions.

### 3.4 Outcome

In the ADVOS registry, a mortality of 60.3% and 64.6% was documented at 28 and 90 days after the first ADVOS treatment session, respectively. Table 6 illustrates the observed mortality rate at each participating center compared to the expected mortality rate according to the median SOFA Score immediately before the first ADVOS session in each patient.

**Table 6:** Predicted mortality vs. documented mortality. Patients discharged from hospital and described as “lost to follow up” before 28 days with unknown outcome at CRF lock were counted as still alive. **01**: Universitätsklinikum Hamburg-Eppendorf (UKE) Zentrum für Anästhesiologie und Intensivmedizin Klinik für Intensivmedizin; **02**: Universitätsmedizin Mainz I. Medizinische Klinik und Poliklinik Johannes-Gutenberg-Universität; **03**: Klinik für Nephrologie Universitätsklinikum Essen; **04**: Kliniken Nordoberpfalz AG Klinikum Weiden.

Site	Predicted mortality	Documented 28-day mortality
01	89.7%	67.2%
02	45.8%	42.9%
03	45.8%	18.2%
04	89.7%	63.2%
Overall	80.0%	60.3%

Finally, a multivariable logistic regression model was developed to identify parameters associated with mortality (Table 7). The model shows that the higher the SOFA Score immediately before the first ADVOS session, the higher is the odds of mortality. The OR was not altered even after correcting the model for age, gender or pre-existing liver disease at admission (data not shown). Other parameters, even if significant in a univariate analysis, did not show significance in the model.

**Table 7:** Multivariable Logistic Regression for parameters associated with 28-day mortality. Variables statistically associated with death in a bivariate analysis were included.

Variable	Odds Ratio	95% CI		Significance
Vasopressors	10.268	0.933	112.992	0.057
Mechanical Ventilation	1.267	0.342	4.693	0.723
Blood pH	0.019	0.000	3.451	0.136
INR	0.699	0.348	1.403	0.313
SOFA Score	1.260	1.056	1.502	0.010^∗^

**Figure 3:**
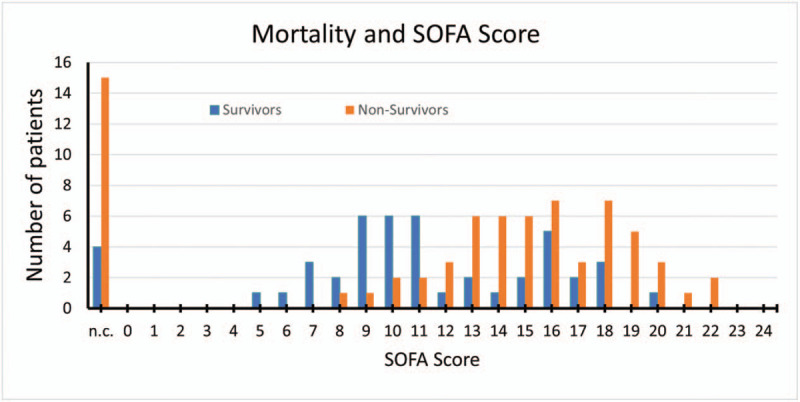
Mortality and SOFA Score before the first ADVOS treatment session among survivors and non-survivors. n.c.: scores not calculated due to missing data.

**Figure 4:**
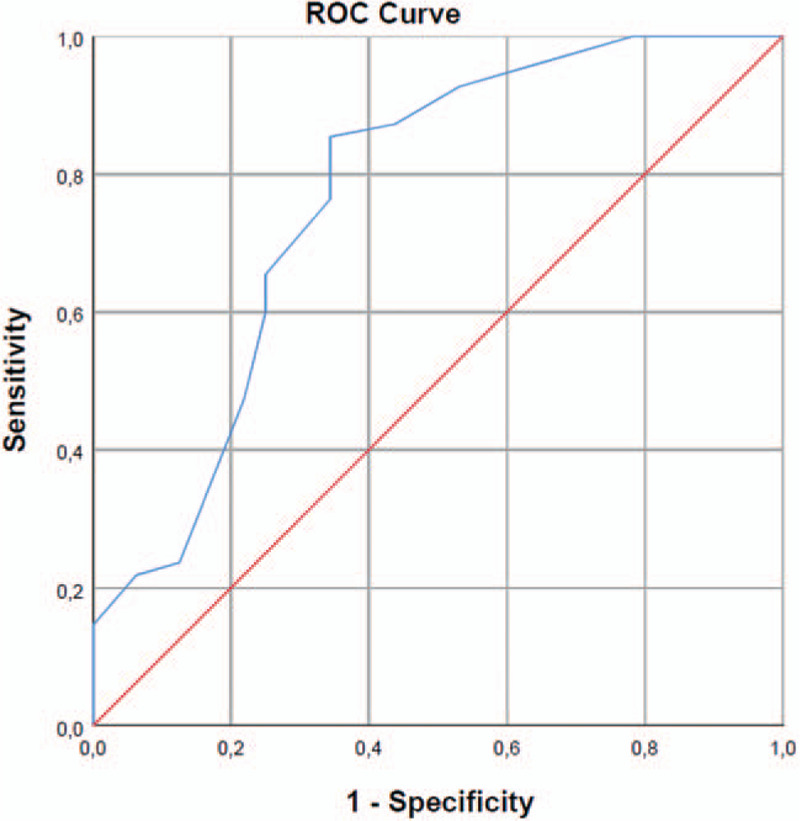
Receiver operating characteristic curve for SOFA Score immediately before the first ADVOS session. Area under the curve (95% CI) = 0.772 (0.672–0.872), *P* < .001; Youden's index at cut-off 11.5 (Sensitivity = 0.855; Specificity = 0.656).

In line with this, among survivors lower SOFA Scores in comparison to non-survivors were observed (11 vs 16), suggesting a better outcome when ADVOS is not used as a last line therapy (Fig. 3). In fact, the cut-off value of the ROC curve was 11.5, with an area under the curve (AUC) of 0.772 (Fig. 4).

## 4 Discussion

Patients in the current registry were provided with ADVOS therapy according to its indications for use. The data summarized in this report provide real world evidence (RWE) on an emerging albumin hemodialysis device for multiple organ support. The obtained RWE is reliable as it holds enough quality based on its complete, transparent, generalizable, timely, and scalable documented data.^[26]^ This is reflected, among others, by the design of the study, the use of a clinical trial management tool provided with edit checks, by the possibility to perform audit trails and by the completeness of the data sheets with more than 80% of the required data fulfilled.

### 4.1 Safety

Less than 6% of the treatments were aborted due to device errors, and only 13 clotting cases were described as adverse events related to the ADVOS therapy, none of them serious. According to the more than 6800 hours of ADVOS treatment in this study, the therapy can be considered safe. The significant reduction of platelet count seems to be not different to common dialysis treatments.^[27,28]^ Moreover, ADVOS therapy runs at low blood flows (median of 120 ml/minutes in this cohort), which reduces the risk of bleeding or hemolysis prevalent during other blood purification therapies, such as extracorporeal membrane oxygenation (ECMO) or extracorporeal CO_2_ removal (ECCO2R) with higher blood flows.^[29,30]^

All the electrolytes remained in the physiological range during treatments (Table 3) but a significant reduction of sodium and chloride occurred. While the decrease of sodium was not clinically relevant (140 vs 138 mmol/l), the reduction of chloride (108 vs 103 mmol/l) might be related to the treatment and correction of hyperchloremic acidosis.

### 4.2 Performance

Three main benefits have been observed with ADVOS: a reduction in protein-bound disease markers (e.g., bilirubin), a removal of water-soluble substances (e.g., creatinine or BUN) and a correction of acid-base parameters (e.g., pH, and HCO_3_^-^). These data correlate with those already presented in the pre-clinical trials,^[16,17]^ and in clinical experiences.^[18–20]^ Newer case-series currently being completed and already presented in scientific meetings show a similar trend.^[22,31]^

This reflects the possibility to simultaneously support 3 organs (i.e., liver, kidney, and lung) with the ADVOS system. Huber recently reported ADVOS to be “the first integrated MOST device” (MOST = multiple organ support therapy).^[32]^ In this review, they were highlighted the specific features of the “intelligent” dialysate of ADVOS, which can be adapted to the patient needs, allowing a correction of acid-base imbalances, including CO_2_ removal.^[20,21]^ A recent publication from his group reported a median CO_2_ removal of 48 ml/minutes in a COVID-19 patient with multiple organ failure.^[33]^

### 4.3 Potential benefit for different patient groups and basis for future studies

The current cohort presents a heterogeneous population including patients with very different diagnosis and prognosis. First, patients with different organ dysfunctions and failures (in combination or alone) including liver, kidney, coagulation, cardiovascular or respiratory systems were recruited (Fig. 2). Furthermore, the SOFA score of these patients immediately before the first ADVOS treatment ranged from 5 to 22 (Fig. 3). Finally, each of the clinics enrolled different type of patients.

Therefore, these results might not be generalizable. In contrast, a subgroup analysis should be performed in order to identify different sub-cohorts. In these subgroups, controlled clinical trials are warranted to define the target population that could benefit most from the ADVOS therapy. Specially interesting could be the case of patients with acid-base disorders. In this report, 52.5% of patients had acidemia, 25% elevated pCO_2_ levels and 55% serum bicarbonate levels below commonly accepted physiological ranges.

### 4.4 Limitations

First, a patient registry lacks the level of evidence of a randomized controlled trial (RCT). However, the real-world evidence obtained with these results are of valuable importance, as discussed previously.

Second, only 118 patients without a control group were included. What is more, the type and number of patients included by each participating site was very different. This makes difficult to draw conclusions, especially in the centers with a lower number of patients. Nevertheless, as shown in Table 6, ADVOS therapy was feasible and results were encouraging in each of the centers.

Third, most of the data were obtained retrospectively. According to the study plan, this did not allow to a complete follow-up (FU) (i.e., visits on days 28 and 90 after the first ADVOS session) unless the patient remained in hospital during the (FU) period. As a result, data on long-term outcome was not obtained.

Fourth, even if the medical history was documented, an exact diagnosis was not reported. This issue hinders a sub-group analysis according to the underlying syndrome. In order to perform such analysis, surrogate parameters such as bilirubin, creatinine, lactate or acid-base parameters could be used instead.^[34–39]^

Finally, as a consequence of the absence of consensus guidelines for the use of ADVOS, patients were probably treated too late. As shown by the high baseline SOFA Score before the ADVOS treatments (median of 14), multiple organ failure had already developed in most of the patients, which significantly reduced the chances of survival.^[9]^ In fact, using a cut-off value of 16, huge differences are shown in mortality rates in our patients (SOFA <16: 47% vs SOFA ≥16: 72%). New studies comparing patients with different baseline severity of illness might be helpful to define a target population who could benefit most from the ADVOS therapy.

## 5 Summary and interpretation

This 2-year report shows that the Registry on Extracorporeal Multiple Organ Support with the ADVOS system is a feasible and valuable tool to increase the evidence on the safety and performance of the ADVOS therapy. On the 1 hand, data on removal of water-soluble and protein-bound substances, as well as acid-base correction correlate well with previous results.^[16–21]^ On the other hand, the small number of device-related adverse events highlights the safety of the therapy. Moreover, a trend towards a mortality rate reduction was observed in each of the participating sites. Nevertheless, all the data provided in this report should be carefully interpreted due to the nature of patient's registries.

## Acknowledgments

We want to acknowledge Prof. Wolfgang Huber for its valuable advice during the study design and the manuscript writing.

## Author contributions

VF and JL participated in the study design and conceptualization. AP and VF contributed with the statistical analysis and wrote the manuscript. AF, DJ, JL, and BT participated in data curation. JWM, AK, and SK provided critical review with important intellectual input. All authors are accountable for all aspects of the work, and all authors read and approved the final manuscript.

**Conceptualization:** Valentin Fuhrmann, Jens Lutz.

**Data curation:** Andreas Faltlhauser, Bartosz Tyczynski, Dominik Jarczak, Jens Lutz, Julia Weinmann–Menke.

**Formal analysis:** Valentin Fuhrmann, Aritz Perez Ruiz de Garibay.

**Funding acquisition:** Aritz Perez Ruiz de Garibay.

**Methodology:** Valentin Fuhrmann.

**Supervision:** Valentin Fuhrmann.

**Writing – original draft:** Valentin Fuhrmann, Aritz Perez Ruiz de Garibay.

**Writing – review & editing:** Valentin Fuhrmann, Aritz Perez Ruiz de Garibay, Andreas Faltlhauser, Bartosz Tyczynski, Dominik Jarczak, Jens Lutz, Julia Weinmann–Menke, Andreas Kribben, Stefan Kluge.

## Supplementary Material

Supplemental Digital Content
