# Supplementary material for: Registry on extracorporeal multiple organ support with the advanced organ support (ADVOS) system: 2-year interim analysis
Source: Medicine (Baltimore). 2021 Feb 19;100(7):e24653. doi: 10.1097/MD.0000000000024653 (PMC7899840; doi:10.1097/MD.0000000000024653)
Supplement: Supplemental Digital Content [file medi-100-e24653-s001.docx]

Supplementary Table 1. Medical History at hospital admission. More than one disease could be selected for each patient.

|  | **Num. of Patients** | **% of Patients** |
| --- | --- | --- |
| **AIDS / HIV** | 1 | 0,8% |
| **Cerebrovascular disease** | 8 | 6,8% |
| **Chronic pulmonary disease** | 17 | 14,4% |
| **Congestive heart failure** | 12 | 10,2% |
| **Dementia** | 1 | 0,8% |
| **Diabetes (with end organ damage)** | 4 | 3,4% |
| **Diabetes (without end organ damage)** | 24 | 20,3% |
| **Hemiplegia or paraplegia** | 2 | 1,7% |
| **Leukemia** | 1 | 0,8% |
| **Lymphoma** | 4 | 3,4% |
| **Malignancy (during last 5 years)** | 22 | 18,6% |
| **Metastatic solid tumour** | 5 | 4,2% |
| **Mild liver disease** | 11 | 9,3% |
| **Moderate or severe liver disease** | 68 | 57,6% |
| **Moderate or severe renal disease** | 24 | 20,3% |
| **Myocardial infarction** | 7 | 5,9% |
| **Peptic ulcer disease** | 10 | 8,5% |
| **Peripheral vascular disease** | 10 | 8,5% |
| **Rheumatologic disease** | 1 | 0,8% |
